# Overexpression of EMMPRIN Isoform 2 Is Associated with Head and Neck Cancer Metastasis

**DOI:** 10.1371/journal.pone.0091596

**Published:** 2014-04-04

**Authors:** Zhiquan Huang, Ning Tan, Weijie Guo, Lili Wang, Haigang Li, Tianyu Zhang, Xiaojia Liu, Qin Xu, Jinsong Li, Zhongmin Guo

**Affiliations:** 1 Department of Oral and Maxillofacial Surgery, Sun Yat-sen Memorial Hospital, Sun Yat-sen University, Guangzhou, Guangdong, China; 2 Guangxi Key Laboratory of Molecular Medicine in Liver Injury and Repair, Guilin Medical College, Guilin, Guangxi, China; 3 Shanghai Medical College, Fudan University, Shanghai and State Key Laboratory of Oncogenes and Related Genes, Shanghai Cancer Institute, Renji Hospital, Shanghai Jiao Tong University School of Medicine, Shanghai, China; 4 Department of Pathology, Sun Yat-sen Memorial Hospital, Sun Yat-sen University, Guangzhou, Guangdong, China; 5 Department of Urinary Surgery, The Affiliated Hospital of Guilin Medical College, Guilin Medical College, Guilin, Guangxi, China; 6 Department of Pathology, The Affiliated Hospital of Guilin Medical College, Guilin Medical College, Guilin, Guangxi, China; 7 Medical Sciences Program, Indiana University School of Medicine, Bloomington, Indiana, United States of America; Johns Hopkins University, United States of America

## Abstract

Extracellular matrix metalloproteinase inducer (EMMPRIN), a plasma membrane protein of the immunoglobulin (Ig) superfamily, has been reported to promote cancer cell invasion and metastasis in several human malignancies. However, the roles of the different EMMPRIN isoforms and their associated mechanisms in head and neck cancer progression remain unknown. Using quantitative real-time PCR, we found that EMMPRIN isoform 2 (EMMPRIN-2) was the only isoform that was overexpressed in both head and neck cancer tissues and cell lines and that it was associated with head and neck cancer metastasis. To determine the effects of EMMPRIN-2 on head and neck cancer progression, we transfected head and neck cancer cells with an EMMPRIN-2 expression vector and EMMPRIN-2 siRNA to exogenously modulate EMMPRIN-2 expression and examined the functional importance of EMMPRIN-2 in head and neck cancer invasion and metastasis. We found that EMMPRIN-2 promoted head and neck cancer cell invasion, migration, and adhesion in vitro and increased lung metastasis in vivo. Mechanistic studies revealed that EMMPRIN-2 overexpression promoted the secretion of extracellular signaling molecules, including matrix metalloproteinases-2(MMP-2), urokinase-type plasminogen activator(uPA) and Cathepsin B, in head and neck cancer cells. While MMP-2 and uPA have been demonstrated to be important mediators of EMMPRIN signaling, the role of Cathepsin B in EMMPRIN-mediated molecular cascades and tumorigenesis has not been established. We found that EMMPRIN-2 overexpression and Cathepsin B down-regulation significantly inhibited the invasion, migration and adhesion of Tca8133 cells, suggesting that Cathepsin B is required for EMMPRIN-2 enhanced cell migration and invasion in head and neck cancer. The results of our study demonstrate the important role of EMMPRIN-2 in head and neck cancer progression for the first time and reveal that increased extracellular secretion of Cathepsin B may be a novel mechanism underlying EMMPRIN-2 enhanced tumor progression in head and neck cancer.

## Introduction

Head and neck cancer (HNC) is the sixth most common cancer worldwide [1], and has become more prevalent in developing countries over the past decade [2]. More than 650,000 new cases of HNC are diagnosed each year worldwide [3], [4]. In Europe alone, approximately 143,000 new cases and greater than 68,000 deaths occur due to the disease each year [4]. Surgery combined with chemotherapy and radiotherapy is now accepted as the most effective treatment for patients with head and neck squamous cell carcinoma. However, the mortality rate due to head and neck cancer has not changed significantly in the past 30 years, and the 5-year survival rate continues to be less than 50% [2]. Treatment failure has mainly been attributed to local recurrence and distant metastasis [5]. At present, therapeutic decisions are made based on clinicopathologic parameters, including age, tumor node metastasis stage, and histologic grade. Although useful, these factors often fail to provide accurate information regarding the biological features of the tumors [3]. Therefore, insight into the molecular alterations associated with head and neck cancer metastasis will provide critical insights into the fundamental mechanisms underlying head and neck cancer progression and further contribute to improvements in the clinical management of head and neck cancer patients.

Extracellular matrix metalloproteinase inducer (EMMPRIN), also known as CD147 or basigin, is a plasma membrane protein of the immunoglobulin (Ig) superfamily and was named based on its function of inducing the production of extracellular matrix metalloproteinases (MMPs), the key enzymes that are involved in maintaining the integrity and turnover of the extracellular matrix (ECM) [6]. EMMPRIN participates in a variety of normal cell physiologies, including lymphocyte responsiveness, female reproductive processes, and intracellular transportation [7]–[9]. Elevated EMMPRIN expression has been correlated with tumor progression in gliomas, giant cell tumors of the bone, laryngeal squamous cell carcinoma, serous ovarian carcinoma and melanomas [10]. EMMPRIN promotes cancer progression by enhancing cancer cell invasion and metastasis. The functional importance of EMMPRIN during tumor progression has mainly been ascribed to its ability to stimulate the production of MMPs [8]–[10]. In tumor cells, EMMPRIN promotes the production of MMP-1, MMP-2, and MMP-9 and facilitates the synthesis of MT1-MMP and MT2-MMP [11]–[13]. In addition to mediating the degradation of the ECM, EMMPRIN plays multifunctional roles in cancer progression. By up-regulating the expression of VEGF and its main receptor, VEGFR-2, in both tumor cells and endothelial cells, EMMPRIN can promote angiogenesis, which is a critical event not only during tumor growth but also during cancer cell metastasis [14], [15]. EMMPRIN can also act as an adhesion molecule and interact with β1-integrin [16], [17]. Many other molecules have been reported to interact with EMMPRIN, including caveolin-1, uPA, monocarboxylate transporters (MCT), and CYP450s [18].

Due to alternative splicing, at least 4 different variants of EMMPRIN mRNA encoding different EMMPRIN protein isoforms (EMMPRIN-1 to -4) have been identified. Among these four variants, EMMPRIN-2 is the most abundant isoform expressed in tumor cells [19]. EMMPRIN-1 encodes the longest, retina-specific isoform, which is distinguished by an additional Ig-like domain (three Ig-like domains in total) in the extracellular portion [20]. The other two variants, EMMPRIN-3 and EMMPRIN-4, were first identified in human endometrial stromal cells and cervical carcinoma cell lines [19]. EMMPRIN-3 is the shortest isoform, consisting of only one Ig-like domain in its extracellular portion [21], and it interacts with the internalized EMMPRIN receptor-ligand complex [19].

Our previous study identified EMMPRIN as an effective target for immunotherapy for tongue squamous cell carcinoma [22]. However, the precise characteristics of the EMMPRIN isoforms and their roles in the initiation and progression of head and neck cancer remain unknown. While EMMPRIN isoform 3 is a demonstrated negative regulator in proliferation and invasion of cancer cells [23], EMMPRIN isoforms 1, 2 and 4 have been suggested to play an oncogenic role in human malignancies including oral cancer. In this study, we investigated the fundamental roles of EMMPRIN isoforms in head and neck cancer progression. Our findings demonstrate the important role of EMMPRIN-2 and its associated mechanisms in head and neck cancer invasion and metastasis for the first time.

## Materials and Methods

### 1. Human tissues and Cell lines

A total of 51 pairs of head and neck cancer tissues and corresponding adjacent nontumorous tissues were collected from 2009 to 2011 at the Department of Oral and Maxillofacial Surgery, Sun Yat-Sen Memorial Hospital, Sun Yat-Sen University, Guangzhou, China. The tissue samples were immediately snap-frozen in liquid nitrogen after resection and stored at −80°C. Both the tumorous and adjacent nontumorous tissues were histologically examined. Written informed consent was obtained for the collection of all human materials, and the study was approved by the Medical Ethics Committee of Sun Yat-sen Memorial Hospital at Zhongshan University. All of the specimens were collected from patients prior to clinical treatment. Clinical features of the patients were summarized in Table 1.

**Table 1:** Clinical characteristics of the patients.

Clinical Characteristic	No. of patients	Percentage
**Age(years)**		
Median	56	
Range	24–88	
**Gender**		
Male	29	56.86%
Female	22	43.14%
**Clinical stages**		
I	0	0.00%
II	14	27.45%
III	12	23.53%
IV	25	49.02%
**T stage**		
1	0	0.00%
2	25	49.02%
3	10	19.61%
4	16	31.37%
**Node status**		
0	25	49.02%
1	10	19.61%
2	16	31.37%
3	0	0.00%
**Metastasis**		
no	25	
yes	26	50.98%
**Differentiation**		
well	33	64.70%
moderate	12	23.53%
poor	5	9.80%
unknown	1	1.96%

The Tca8113 and ACCM head and neck cancer cell lines were kindly provided by the College of Stomatology, Shanghai Jiao Tong University (Shanghai, China) [24], [25]. TSCCA cells were kindly provided by the Sun Yat-Sen University Cancer Center [26] and the source of SCC25 cells was described in our previous publication [27]. The Tca8113 and ACCM cell lines were cultured in RMPI-1640 medium (Invitrogen, Carlsbad, CA), while the TSCCA3 and SCC25 cell lines were cultured in DMEM (Invitrogen Carlsbad, CA). All media were supplemented with 10% fetal bovine serum (Gibco), 100 IU/mL of penicillin G and 100 µg/mL of streptomycin sulfate (Sigma-Aldrich, St. Louis, MO), and the cells were grown in 37°C incubators containing 5% CO_2_ and a humidified atmosphere.

### 2. Reverse transcription and quantitative real-time PCR

Total RNA was extracted from the tissues or cells using TRIzol reagent (Invitrogen) according to the manufacturer's recommended protocol. Complementary DNA was synthesized with the Prime-Script RT Reagent Kit (TaKaRa, Dalian, China). Quantitative RT-PCR (qRT-PCR) analyses were performed with SYBR Premix Ex-Taq (TaKaRa). The primers used are shown in Table S1.

### 3. Western blotting

The cells were lysed in NP-40 lysis buffer containing a protease inhibitor cocktail and a phosphatase inhibitor cocktail (Roche, Rotkreuz, Switzerland). After separation using SDS–PAGE, the proteins were transferred onto nitrocellulose membranes (Bio-Rad, Hercules, USA). Subsequently, the membranes were blocked with 5% non-fat milk in Tris-buffered saline (TBS) containing 0.1% Tween-20 for 1 hour at room temperature. The blots were probed with the relevant primary antibodies overnight at 4°C, washed, and probed with a species-specific horseradish peroxidase-conjugated secondary antibody (Cell Signaling, Beverly, USA). An enhanced chemiluminescence detection method (Pierce ECL Western Blotting Substrate, Thermol, Beverly, MA, USA) was used to visualize the blots. The primary antibodies used were anti-EMMPRIN, anti-MMP-2, anti-uPA, anti-Cathepsin B (Santa Cruz Biotechnology, USA), and anti-β-actin (Sigma–Aldrich, MO, USA).

### 4. EMMPRIN-2 Plasmid Constructs, transfection, lentivirus production and establishment of stably expressing cell lines

The EMMPRIN-2 lentiviral expression vector pWPXL-EMMPRIN-2 was constructed by replacing the GFP fragment of the pWPXL vector (Addgene plasmid 12257, Didier Trono) with the coding sequence of EMMPRIN-2 (accession number NM_198589), which was amplified from the human liver cDNA library using EcoRI and BamHI as cloning enzymes. Oligonucleotides were synthesized to generate annealing shRNAs that targeted the sequence of EMMPRIN-2 from position +430 to +449 (5′-GTCGTCAGAACACATCAAC-3′) and the sequence of Cathepsin B from position +670 to +689 (5′-GTGGCCTCTATGAATCCCA-3′). The sequences of the oligonucleotides for the plasmid constructs are listed in Table S2. The fragments were cloned separately into the pLVTHM vector (Addgene plasmid 12247, Didier Trono) using the MluI and ClaI restriction sites. Lentiviral particles were harvested 48 hours after co-transfection of pWPXL-EMMPRIN-2 or pLVTH-shRNA with psPAX2 and pMD2.G into HEK-293T cells using Lipofectamine 2000 Transfection Reagent (Invitrogen). Target cells (Tca8113 and ACCM) were transduced with recombinant lentivirus plus 6 µg/mL of polybrene (Sigma-Aldrich). After 2 weeks of culture in a 37°C incubator containing 5% CO_2_ and a humidified atmosphere, total RNA and proteins were extracted and the expression of EMMPRIN-2 or Cathepsin B was further verified using western blot and quantitative RT-PCR [28].

### 5. In vitro migration and invasion assays

For the Transwell migration assays, 2×10^4^ cells were plated on the top chamber of each insert (BD Biosciences, NJ). For the invasion assays, 1×10^5^ cells were plated on the upper chamber of each insert, which had been coated with 150 µg of Matrigel (BD Biosciences, MA). The cells in both assays were trypsinized and resuspended in DMEM, and 700–900 µL of medium that had been supplemented with 10% fetal bovine serum was added to the lower chambers of each well. After 12 hours of incubation at 37°C, the cells remaining in the top chambers or on the upper membranes of the inserts were carefully removed, and the cells that had migrated or invaded into the lower chambers were fixed and stained with a dye solution containing 0.1% crystal violet and 20% methanol. The migrating and invading cells were imaged and counted using an IX71 inverted microscope (Olympus, Tokyo, Japan). The same experiments were carried out a minimum of three times.

### 6. Cell Adhesion Assay

A cell adhesion assay was performed according to the protocol that was outlined in a previous publication [29]. For this assay, 96-well flat-bottom culture plates were coated with 30 µg of Matrigel (BD Biosciences, MA) in DMEM for 3 hours at 37°C. Plates that had been coated with 0.2% bovine serum albumin (BSA) for 2 hours at room temperature served as negative controls. The cells were harvested using trypsin/EDTA, washed twice with PBS and resuspended in DMEM. Cells (2.5×10^4^) were added to each well and incubated at 37°C for 30 min. The plates were shaken at 1,000 rpm for 30 seconds and washed three times with DMEM to remove unbound cells. The cells that remained attached to the plates were quantitated using the Cell Counting Kit-8 (CCK-8) Assay (Dojindo Laboratories, Kumamoto, Japan) according to the manufacturer's recommended instructions. After subtraction of background cell binding from BSA-coated wells, the percentage of adherent cells was calculated by dividing the optical density of the adherent cells by that of the initial cell input. Finally, cells that had been fixed and stained in a dye solution containing 0.1% crystal violet and 20% methanol were imaged using an IX71 inverted microscope (Olympus). All of the adhesion experiments were conducted in triplicate wells and repeated a minimum of three times.

### 7. Mouse models for in vivo analyses of metastasis

For the *in vivo* analyses of the metastatic capacities of the cell lines, each nude mouse (ten per group, male BALB/c-nu/nu) was tail-intravenously injected with 1×10^6^ Tca8113 cells stably expressing EMMPRIN-2 or Tca8113 cells expressing the empty vector. The mice were sacrificed after 6 weeks, and the lungs were dissected, fixed with phosphate-buffered neutral formalin and paraffin embedded. Metastatic foci in the lungs were counted microscopically on H&E-stained tissue sections. The mice were manipulated and housed in accordance with protocols that had been approved by the Shanghai Medical Experimental Animal Care Committee and the study was approved by the Research Ethics Committee of Shanghai Medical College, Fudan University.

### 8. Gelatin zymography assay

The gelatin zymography assay was performed to determine the activity of MMP-2 [30]. Briefly, the cells were incubated in serum-free medium, and the medium was collected after 24 hours. Medium from an equal number of cells was separated using 10% acrylamide gels containing 1 mg/ml of gelatin type A (Sigma–Aldrich). The gels were incubated in a 2.5% Triton-X-100 solution at room temperature with gentle agitation to remove SDS and re-nature the MMP-2. The gels were then incubated in developing buffer (50 mM Tris-HCl, 0.2 M NaCl, 5 mM CaCl_2_, and 0.02% Brij35) for 24 hours at 37°C to induce gelatin lysis by the renatured MMP-2. After reaction, the gels were stained with staining solution (0.1% Coomassie Brilliant Blue R250, 30% methanol, and 10% acetic acid) for 1 hour and destained in a solution containing 30% methanol and 10% acetic acid. Images of the gels were acquired using the ChemiDoc XRS+ System (Bio-Rad). The same experiments were carried out a minimum of three times.

### 9. ELISA assays

Sample preparation: Cells (2×10^6^) were added to each 10 cm dish and cultured for 24 hours. To remove the unbound cells, the dishes were washed three times with serum-free medium. Next, the cells were incubated with 15 ml of serum-free media, and the media was collected using an Amicon Ultra 15 ml 10K column (Millipore) after 24 hours. uPA activity, MMP-2 and Cathepsin B concentrations were quantified using a uPA Activity Assay Kit (Millipore), a Human MMP-2 Quantikine ELISA Kit (R&D Systems) and a Human Cathepsin B Duo Set Kit (R&D Systems) according to the manufacturers recommended protocols. All of the sample analyses were performed in triplicate and repeated a minimum of three times.

### 10. Statistical analyses

The results are presented as the mean +/− the standard error of the mean (SEM). Differences between groups were assessed using either one-way analysis of variance (ANOVA) or a two-tailed Student's t-test, as specified in the corresponding figure legends. *P*<0.05 was set as the level of statistical significance. All of the statistical analyses were performed using SPSS 17.0.

## Results

### 1. EMMPRIN-2 is overexpressed in head and neck cancer and is associated with head and neck cancer metastasis

**Figure 1:**
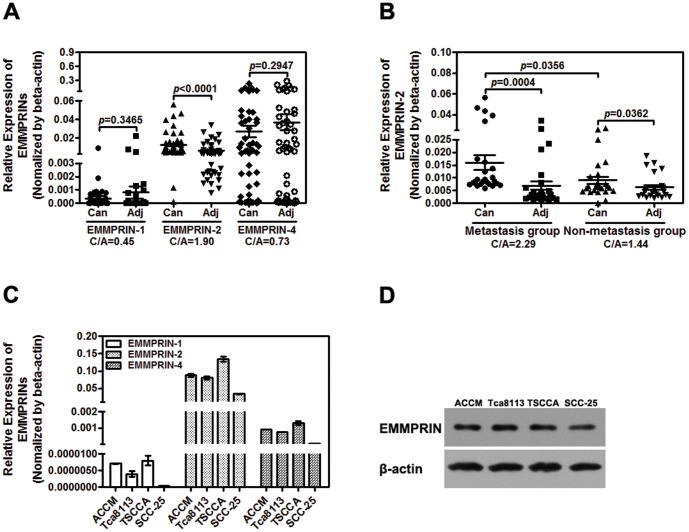
EMMPRIN-2 is overexpressed in head and neck cancer and is associated with head and neck cancer metastasis. (A) EMMPRIN mRNA expression in 51 head and neck cancer tissues and the adjacent non-cancerous tissues was analyzed using qPCR. EMMPRIN-2 mRNA expression in head and neck tumor tissues is higher than that in adjacent non-cancerous tissues (*p*<0.01). No significant differences were observed in EMMPRIN-1 and EMMPRIN-4 mRNA expression between tumor tissues and the adjacent non-cancerous tissues (*p*>0.05). (B) Comparison of EMMPRIN-2 mRNA expression between 26 cases of metastatic tumors and 25 cases of tumors without any sign of local and distant metastases. EMMPRIN-2 mRNA expression was significantly increased in metastatic head and neck cancer tissues compared to non-metastatic head and neck cancer tissues (*p*<0.05). (C) qPCR detection of EMMPRIN mRNA expression in head and neck cancer cell lines. (D) Detection of EMMPRIN expression in head and neck cancer cell lines using Western blot. High levels of protein encoded by the EMMPRIN-2 gene were detected in all 4 of the cancer cell lines.

Although altered expression of various EMMPRIN isoforms has been implicated to play a role in HNC tumorigenesis, the individual contributions of the different EMMPRIN isoforms to the initiation and progression of HNC remain unclear. To further elucidate the roles of the EMMPRIN isoforms in HNC, we examined the expression of different EMMPRIN isoforms in HNC tissues and cell lines. The expression of all 4 EMMPRIN isoforms was first examined in 12 cases of oral cancer tissues using quantitative real-time PCR. While EMMPRIN isoforms 1, 2 and 4 showed relatively abundant expression, EMMPRIN isoform 3 was hardly detectable in the tested oral cancer tissues (Figure S1). Therefore, our continuing study focused on the roles of EMMPRIN isoforms 1, 2 and 4 in head and neck cancer progression. The expression of EMMPRIN isoforms 1, 2 and 4 was further analyzed in 51 HNC tumors and their matched normal oral tissues using quantitative RT-PCR. As shown in Figure 1A, EMMPRIN-2 and EMMPRIN-4 appeared to be the main isoforms that were expressed in head and neck tissues, while EMMPRIN-1 displayed only very low levels of expression. We observed that EMMPRIN-2 was the only EMMPRIN isoform in which the mRNA expression levels were significantly increased in the tumors compared to the matched adjacent non-cancerous tissues (Fig. 1A). Approximate 2-fold increases (C/A, 1.90) in average EMMPRIN-2 expression levels were detected in tumor tissues over the adjacent normal controls. No significant differences in EMMPRIN-1 and EMMPRIN-4 mRNA expression were detected between the tumors and the adjacent non-cancerous tissues (Fig. 1A).

To further determine the association between EMMPRIN-2 expression and the progression and metastasis of HNC, we compared EMMPRIN-2 mRNA expression in 26 cases of metastatic tumors and 25 cases of tumors without any sign of local and distant metastasis. While marginally increased EMMPRIN-2 expression was detected in tumors compared to the adjacent normal tissues from patients with non-metastatic tumors (Fig. 1B), marked differences in EMMPRIN-2 expression were observed between tumors and the normal control tissues from patients with metastatic tumors (Fig. 1B). Moreover, EMMPRIN-2 expression was significantly higher in metastatic tumors than in non-metastatic tumors (Fig. 1B). No significant differences in the gender or age of patients with metastatic and non-metastatic tumors were found.

Findings from the HNC tissues were further confirmed by the analyses conducted in the HNSCC cell lines (ACCM, Tca8113, TSCCA and SCC25). EMMPRIN-2 and EMMPRIN-4 were the major isoforms that were expressed in all 4 cell lines, and the relative expression levels of EMMPRIN-2 were much higher in the cancer cell lines (ranging from 0.05 to 0.15, Fig. 1C) than in the non-cancerous HNC tissues (which averaged approximately 0.01, Fig. 1A). High levels of protein that were encoded by the EMMPRIN-2 gene were also detected in all 4 cancer cell lines using western blot (Fig. 1D).

### 2. EMMPRIN-2 promotes head and neck cancer cell invasion, migration, and adhesion in vitro, and metastasis in vivo

**Figure 2:**
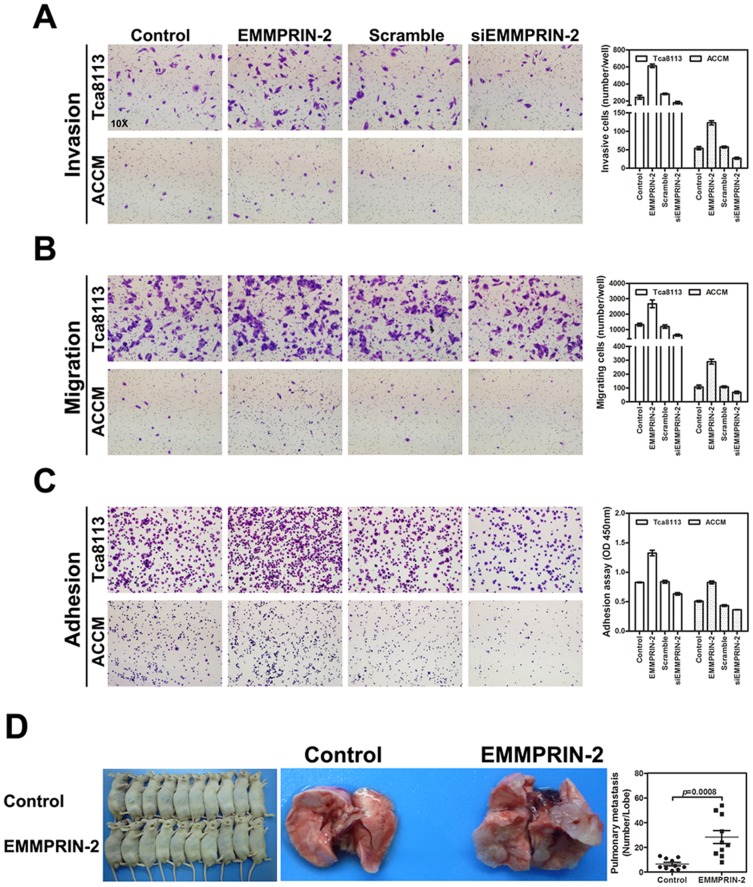
EMMPRIN-2 expression modulated head and neck cancer cell invasion, migration, and adhesion in vitro, and metastasis in vivo. Exogenous overexpression of EMMPRIN-2 using an expression construct enhanced head and neck cancer cell invasion (A), migration (B) and adhesion (C), whereas down-regulation of EMMPRIN-2 using siRNA (siEMMPRIN-2) suppressed head and neck cancer cell invasion (A), migration (B) and adhesion (C). The differences in cell invasion, migration and adhesion between the construct- or siRNA-transfected groups and the mock controls were statistically significant (*p*<0.05). The effects of EMMPRIN-2 expression on tumor metastasis in vivo were further investigated in nude mouse models. (D) Tca8113 head and neck cancer cell lines with stably expressed EMMPRIN-2 or a mock control vector were intravenously injected into nude mice. Metastatic foci in the lungs of nude mice were calculated at week 6 after injection. The average number of metastatic foci in the lungs from the mice with the EMMPRIN-2 expressing cells was 28.4 compared to 6.4 in the lungs from the mice injected with cells bearing the control vector (*p*<0.01).

To determine the functional roles of EMMPRIN-2 over-expression in the invasion, migration and adhesion of HNC cells, we experimentally over-expressed and down-regulated EMMPRIN-2 expression using an exogenous EMMPRIN-2 expression vector and siRNA (siEMMPRIN-2) in the head and neck cell lines, ACCM and Tca8113. As shown in Figure 2, enhanced expression of EMMPRIN-2 promoted head and neck cancer cell invasion, adhesion and migration in both the ACCM and Tca8113 cell lines. In contrast, experimental down-regulation of EMMPRIN-2 using siRNA attenuated cell invasion, migration and adhesion in the tested cell lines (Figs. 2A, B and C).

We further extended our in vitro observations to examine the effects of EMMPRIN-2 on HNC cell metastasis in vivo. Tca8113 cells stably over-expressing EMMPRIN-2 or the control vector were intravenously injected into nude mice through the tail vein. The mice were sacrificed 6 weeks after the injections and the lungs were dissected, fixed with phosphate-buffered neutral formalin and paraffin-embedded. Metastatic foci in the lungs of the nude mice were counted microscopically on H&E stained tissue sections. Significantly higher numbers of metastatic foci were observed in the lungs of mice that had been injected with Tca8113 cells expressing EMMPRIN-2. The average number of metastatic foci in the lungs from the mice with the EMMPRIN-2 expressing cells was 28.4, compared to 6.4 in the lungs from the mice that had been injected with the Tca8113 cells bearing the control vector (Fig. 2D).

### 3. EMMPRIN-2 overexpression promotes the secretion of the extracellular signaling molecules MMP-2, uPA and Cathepsin B in head and neck cancer cells

**Figure 3:**
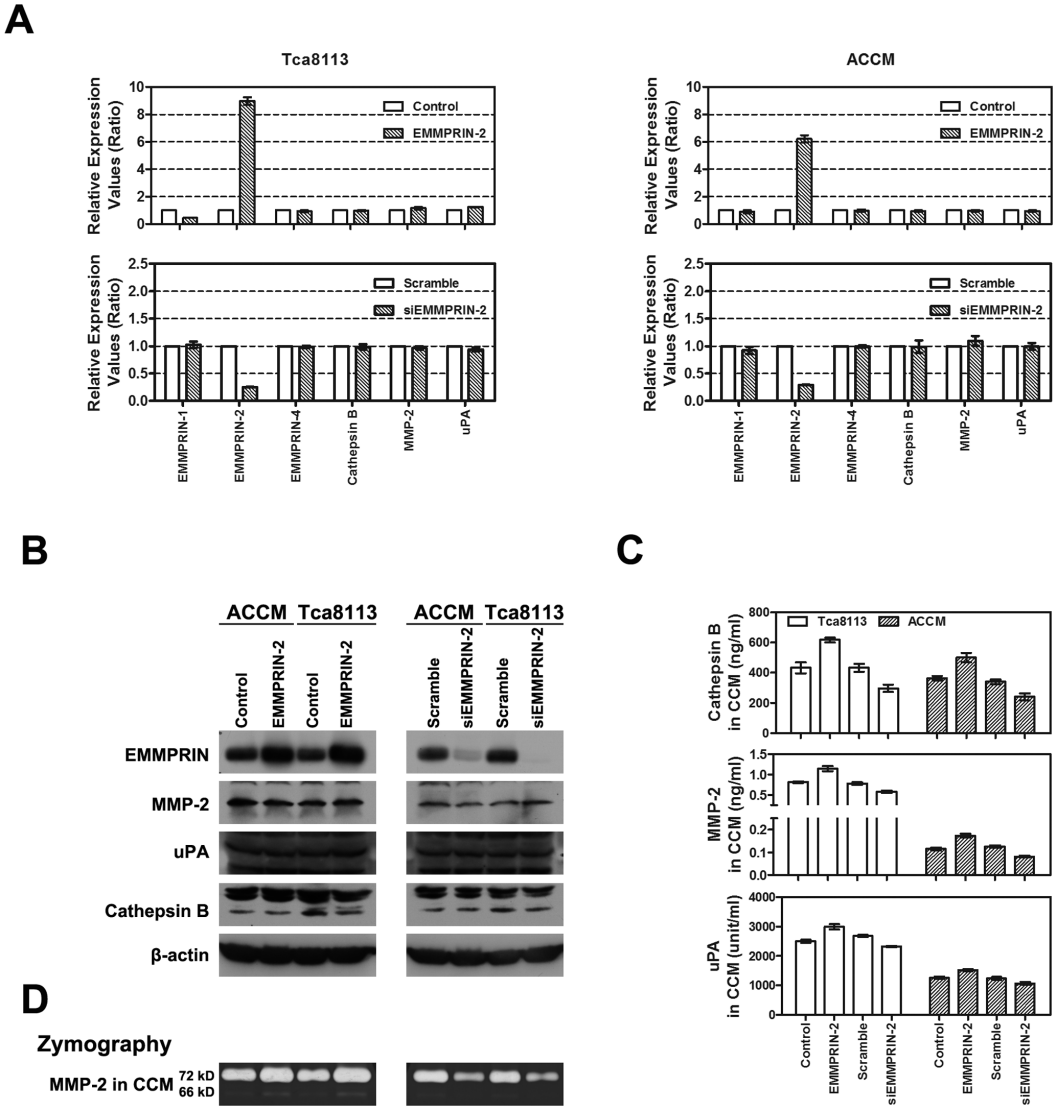
Overexpression of EMMPRIN-2 promotes the secretion of MMP-2, Cathepsin B and uPA in head and neck cancer cells. (A) The mRNA expression levels of uPA, Cathepsin B, MMP-2 and different EMMPRIN isoforms in head and neck cancer cells were examined after exogenous expression of EMMPRIN-2. Transfection of an EMMPRIN-2 expression vector or siRNA (siEMMPRIN) selectively enhanced or attenuated the mRNA expression levels of EMMPRIN-2 in both ACCM and Tca8113 cells, while the expression of other EMMPRIN isoforms, uPA, Cathepsin B and MMP-2 remained unaffected (*p*>0.05). (B) The mRNA expression findings were confirmed by analyzing protein expression using western blot. (C) Detection of extracellular MMP-2, Cathepsin B and uPA using ELISA. MMP-2, Cathepsin B and uPA secretion into the culture medium was increased in cells that had been transfected with the EMMPRIN-2 expression vector, while secretion was decreased in EMMPRIN-2 siRNA transfected cells (*p*<0.05). (D) Analysis of extracellular MMP-2 activity using a gelatin zymography assay. Extracellular MMP-2 activity was increased after EMMPRIN-2 expression was enhanced, while MMP-2 activity was reduced after EMMPRIN-2 expression knockdown.

EMMPRIN has been well demonstrated to enhance cancer cell invasion in several human malignancies by increasing the expression of the extracellular signaling molecules MMP-2 and uPA [6], [10], [31], [32]. In addition, Cathepsin B, a member of the lysosomal proteolytic enzyme Cathepsin family, has also been reported to play crucial roles in cancer invasion and metastasis [33]. To identify the underlying mechanisms governing the effects of EMMPRIN-2 on cancer cell invasion and metastasis, we examined the expression and secretion of uPA, Cathepsin B and MMP-2 in head and neck cancer cells after exogenously modulating the expression of EMMPRIN-2. Transfection of an EMMPRIN-2 expression vector or siRNA (siEMMPRIN) selectively enhanced or attenuated the expression of EMMPRIN-2 at the mRNA level in both the ACCM and Tca8113 cells, while the expression of the other EMMPRIN isoforms, uPA, Cathepsin B and MMP-2 remained unaffected (Fig. 3A). Similar results were observed when protein expression was detected using western blotting (Fig. 3B).

In addition, we examined the extracellular levels of all three proteins using ELISA. The extracellular levels of MMP-2, uPA and Cathepsin B in the cell culture medium were increased by EMMPRIN-2 expression vector transfection and were decreased by EMMPRIN-2 siRNA transfection in both ACCM and Tca8113 cells (Fig. 3C). These findings suggest that EMMPRIN-2 specifically promotes the extracellular secretion, rather than the expression, of uPA, MMP-2 and Cathepsin B in head and neck cancer cells. The effects of EMMPRIN-2 on MMP-2 extracellular secretion were further verified using a gelatin zymography assay. As shown in Figure 3D, extracellular MMP-2 activity increased in both ACCM and Tca8113 cells that had been transfected with the EMMPRIN-2 expression vector, whereas extracellular MMP-2 activity decreased in the cell lines that had been transfected with EMMPRIN-2 siRNA (Fig. 3D).

### 4. Cathepsin B is an important mediator of the effects of EMMPRIN-2 on the invasion, migration and adhesion of head and neck cancer cells

**Figure 4:**
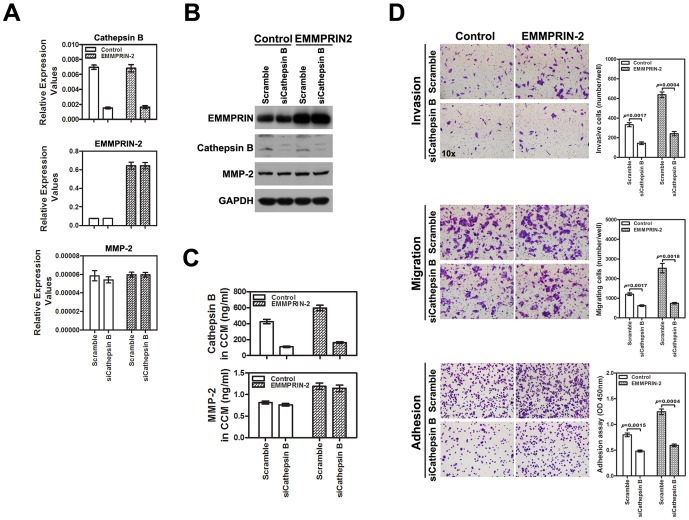
Cathepsin B is required for EMMPRIN-2-mediated invasion, migration and adhesion of head and neck cancer cells. (A) Treatment with Cathepsin B siRNA resulted in corresponding reductions in Cathepsin B mRNA levels in both EMMPRIN-2 overexpressing and parental Tca8113 cells (*p*<0.05), while EMMPRIN-2 and MMP-2 were unaffected by Cathepsin B siRNA transfection (*p*>0.05). (B) The effects on Cathepsin B, EMMPRIN-2 and MMP-2 expression following Cathepsin B siRNA transfection in EMMPRIN-2 overexpressing and parental Tca8113 cells were further confirmed using western blot analyses. (C) The extracellular secretion of Cathepsin B was also reduced by siRNA treatment in Tca8113 cells both in the presence and absence of EMMPRIN-2 overexpression (*p*<0.05). (D) As observed in the experiments above, Tca8113 cells overexpressing EMMPRIN-2 displayed increased cell invasion, migration and adhesion as compared to parental Tca8113 cells. In contrast, down-regulation of Cathepsin B using siRNA significantly inhibited the invasion, migration and adhesion of Tca8113 cells overexpressing EMMPRIN-2.

To determine the functional role of Cathepsin B in EMMPRIN-2-induced invasion, migration and adhesion, we down-regulated Cathepsin B expression in Tca8113 cells overexpressing EMMPRIN-2 using Cathepsin B siRNA. Treatment with Cathepsin B siRNA resulted in corresponding reductions in Cathepsin B mRNA and protein levels in both the parental and EMMPRIN-2 overexpressing Tca8113 cells, while EMMPRIN-2 and MMP-2 were unaffected by Cathepsin B transfection (Figs. 4A and B). Extracellular secretion of Cathepsin B was also reduced by siRNA treatment in Tca8113 cells, both in the presence and absence of EMMPRIN-2 overexpression (Fig. 4C). As observed in the experiments outlined above, Tca8113 cells overexpressing EMMPRIN-2 displayed increased cell invasion, migration and adhesion compared to the parental Tca8113 cells (Figure 4D). In contrast, down-regulation of Cathepsin B using siRNA significantly inhibited the invasion, migration and adhesion of Tca8113 cells overexpressing EMMPRIN-2 (Fig. 4D), implicating the functional importance of Cathepsin B in EMMPRIN-2-mediated signaling for tumor invasion and metastasis.

## Discussion

Despite the progress that has been made in the treatment of locally advanced head and neck cancer, the prognosis remains dismal, and 5-year survival does not exceed 40% [34]. Local recurrence and metastasis are responsible for the majority of deaths due to head and neck cancer. Although the molecular and genetic factors contributing to head and neck cancer progression are not completely understood, the altered expression of molecules that are involved in cell signaling and interactions between cancer cells and the stroma has been proposed as an important mechanism underlying head and neck cancer invasion and metastasis [35].

EMMPRIN is a transmembrane protein family that regulates the turnover and remodeling of the extracellular matrix (ECM) and is an important mediator of cell and stromal interactions [6], [10]. Four different isoforms are encoded by the gene and 3 isoforms (EMMPRIN-1, -2 and -4) have been identified to be dominantly expressed in epithelial cells. By enhancing the extracellular secretion of MMPs, VEGF and VEGFR-2 and their interactions with other plasma membrane proteins, EMMPRIN accelerates the degradation of the ECM and promotes the migration, invasion and metastasis of cancer cells [11]–[17]. Overexpression of EMMPRIN proteins has been reported in several human malignancies and has been found to be associated with the invasion and metastasis of the tumors [10], [11]. Furthermore, higher levels of EMMPRIN expression were demonstrated to be correlated with more advanced clinical features and to predict diminished survival in patients with pancreatobiliary adenocarcinomas and ovarian carcinoma [36], [37]. Although abnormal EMMPRIN gene expression has been reported in head and neck cancer [38], the roles of the EMMPRIN gene in the metastasis of head and neck cancer remain unclear. Specifically, the contributions of the different EMMPRIN isoforms to the initiation and progression of head and neck cancer have not been characterized to date. We previously demonstrated that EMMPRIN expression was significantly associated with tumor size, clinical stage, and poor prognosis in head and neck cancer patients. The results of our previous study also implicated the EMMPRIN protein as a promising therapeutic target for patients with salivary gland tumors [39]. In the present study, we extended our previous findings to further investigate the association between EMMPRIN and head and neck cancer invasion and metastasis. Using quantitative RT-PCR and isoform-specific antibodies, we analyzed the expression of the different EMMPRIN isoforms in head and neck cancer tissues and cell lines. Our current study demonstrated for the first time that EMMPRIN isoform 2 (EMMPRIN-2) was the major isoform that was overexpressed in head and neck cancer tissues and was correlated with clinical metastasis of head and neck cancer.

The functional roles of EMMPRIN proteins in tumor invasion and metastasis have been well documented in several human malignancies [10], [36], [37]. However, evidence supporting EMMPRIN as an important driver of human malignancies is still lacking. In addition to the clinicopathological correlation with head and neck cancer metastasis, our study further revealed the functional importance of EMMPRIN-2 in promoting the invasion and metastasis of head and neck cancer cells. Using cell lines stably expressing EMMPRIN-2 protein and siRNA approaches, we demonstrated that EMMPRIN-2 enhanced the migration, adhesion and invasion of head and neck cancer cells in vitro and increased lung metastasis in vivo. This evidence, as well as the findings from primary tumors, suggests that EMMPRIN-2 overexpression is an important mechanism contributing to head and neck cancer invasion and metastasis.

EMMPRIN proteins are extracellular matrix metalloproteinase inducers that are involved in many physiological and pathological states and regulate several distinct molecular pathways. However, the major function of EMMPRIN proteins is to stimulate the synthesis of the extracellular matrix metalloproteinase family. EMMPRIN proteins have been well documented to stimulate the synthesis of MMPs such as MMP-1, MMP-2, MMP-3, and MMP-9 in fibroblast and tumor cells [11], [12]. In addition, uPA has been reported to be an additional molecule that is important in mediating EMMPRIN-associated tumor progression [31], [32]. uPA is ubiquitously expressed in many normal and malignant cell types and is an important component of the fibrinolytic system, converting plasminogen to the active enzyme, plasmin [32]. uPA can be extracellularly secreted, and binding of uPA with its membrane receptor, uPAR, activates down-stream signaling, thereby enhancing angiogenesis, and tumor growth and metastasis [40]. To further understand the molecular mechanisms underlying EMMPRIN-2-associated tumor progression, we examined the effects of EMMPRIN-2 expression on the transcription and extracellular secretion of MMP-2 and uPA in head and neck cancer cells using EMMPRIN-2 stable expression clones and an siRNA-based approach. Contrary to previous findings from other human malignancies, the expression of MMP-2 and uPA at both the mRNA and protein levels was not significantly affected by the exogenous over-expression or siRNA-induced down-regulation of EMMPRIN-2 (Figs. 3A and B). Instead, the extracellular secretion of MMP-2 and uPA was increased by EMMPRIN-2 overexpression and decreased by siRNA knockdown of EMMPRIN-2 expression (Fig. 3C). Regulation of MMP-2 secretion by EMMPRIN-2 in head and neck cancer cells was further supported by corresponding changes in extracellular MMP-2 enzyme activity in EMMPRIN-2 overexpressing cell clones and siRNA transfected cells (Fig. 3D). These findings suggest that increased secretion, rather than the expression of extracellular matrix signaling molecules, is a novel mechanism underlying EMMPRIN-induced tumor invasion and metastasis.

An interesting finding from this study was the identification of Cathepsin B as a downstream mediator of EMMPRIN-2 signaling during the migration and invasion of head and neck cancer cells. Cathepsin B is a member of the lysosomal proteolytic enzyme Cathepsin family. Like other Cathepsin members, Cathepsin B is synthesized as an inactive pre-proenzyme and undergoes post-translational glycosylation. It primarily acts as an intracellular cysteine protease in the lysosome and can be released from the lysosome into the cytoplasm [41]. A number of studies have demonstrated the crucial roles of Cathepsin B in the apoptosis, invasion and metastasis of a variety of human tumors. Cathepsin B has been reported to be associated with lymphatic metastasis in inflammatory breast cancer and is a prognostic marker in colorectal carcinoma [42], [43]. In vitro functional studies revealed that Cathepsin B could be secreted into extracellular compartments [33] and that Cathepsin B mediated the effects of ErbB2 and S1p in breast and prostate cancer cell invasion [44], [45]. In addition, inhibition of Cathepsin B has been shown to attenuate cancer cell invasion and metastasis [46], [47]. Although participation of Cathepsin B in tumor initiation and progression has been suggested in several human malignancies, its fundamental importance in head and neck cancer has not been reported. Our current study provided strong evidence indicating that Cathepsin B functions as an important mediator of EMMPRIN-2 induced signaling, thereby promoting cell migration and invasion in head and neck cancer. We showed that EMMPRIN-2 enhanced the extracellular secretion of Cathepsin B rather than enhancing its transcription, as evidenced by the increased extracellular levels of Cathepsin B and its mRNA and protein levels remained unaltered (Figs. 3A, B and C). More importantly, we observed that the siRNA-mediated down-regulation of Cathepsin B significantly attenuated the EMMPRIN-2-induced migration, adhesion and invasion of head and neck cancer cells (Fig. 4). These findings revealed the importance of the interplay of Cathepsin B and EMMPRIN-2 during invasion and metastasis for the first time and thus provided important insight into the molecular mechanisms underlying head and neck cancer progression.

## Conclusions

The results of this study demonstrated that overexpression of EMMPRIN isoform 2 is a frequent and important event in head and neck cancer invasion and metastasis, and revealed that the increased extracellular secretion of Cathepsin B may be a novel mechanism underlying EMMPRIN-2 enhanced tumor progression in head and neck cancer.

## Supporting Information

Figure S1 **Expression of EMMPRIN isoforms 1, 2, 3 and 4 in primary oral cancer tissues.** mRNA expression of all 4 EMMPRIN isoforms in 12 oral cancer tissues was examined by quantitative real-time PCR.
(TIF)

Table S1 **The sequences of the primers for Quantitative Real-Time Polymerase Chain Reaction.**
(DOCX)

Table S2 **The sequences of the oligonucleotides for plasmids constructs.**
(DOCX)
