# Supplementary material for: Overexpression of EMMPRIN Isoform 2 Is Associated with Head and Neck Cancer Metastasis
Source: PLoS One. 2014 Apr 4;9(4):e91596. doi: 10.1371/journal.pone.0091596 (PMC3976259; doi:10.1371/journal.pone.0091596)
Supplement: Table S1 — The sequences of the primers for Quantitative Real-Time Polymerase Chain Reaction. (DOCX) [file pone.0091596.s002.docx]

Supplementary Table 1. The sequences of the primers for Quantitative Real-Time Polymerase Chain Reaction

| **Primer** | **Sequences** |
| --- | --- |
| EMMPRIN-1-RT-F | 5- CTGCGCTGTTCGTGCTGCTG |
| EMMPRIN-1-RT-R | 5- CGTTGGGACCCTGCCCTTCA |
| EMMPRIN-2-RT-F | 5- CTGCTGGGATTCGCGCTGCT |
| EMMPRIN-2-RT-R | 5- CGCGTCCTCCTTCAGCACCA |
| EMMPRIN-3-RT-F | 5-TGCTGAGAGTCTGGGTTTACG |
| EMMPRIN-3-RT-R | 5-TGGAGCCAAGGTCTTCTACG |
| EMMPRIN-4-RT-F | 5- CCAAGAGACGCCCCCACCTG |
| EMMPRIN-4-RT-R | 5- GGGAGACGCGTCCGACTGCT |
| Beta-actin-RT-F | 5- AGTGTGACGTGGACATCCGCAAAG |
| Beta-actin-RT-R | 5- ATCCACATCTGCTGGAAGGTGGAC |
| MMP-2-RT-F | 5- CGGGCCTGGAGAACTAGAGAAGGAC |
| MMP-2-RT-R | 5- GCCTGGGAGGAGTACAGTCAGCATC |
| uPA -RT-F | 5- CGCAGTCACACCAAGGAAGAGAATG |
| uPA -RT-R | 5- TCTGTGCAGAGCCTATCTTCCCAGT |
| Cathepsin B-RT-F | 5- CCAAGAGACGCCCCCACCTG |
| Cathepsin B-RT-R | 5- GGGAGACGCGTCCGACTGCT |
