# Supplementary material for: Overexpression of EMMPRIN Isoform 2 Is Associated with Head and Neck Cancer Metastasis
Source: PLoS One. 2014 Apr 4;9(4):e91596. doi: 10.1371/journal.pone.0091596 (PMC3976259; doi:10.1371/journal.pone.0091596)
Supplement: Table S2 — The sequences of the oligonucleotides for plasmids constructs. (DOCX) [file pone.0091596.s003.docx]

Supplementary Table 2. The sequences of the oligonucleotides for plasmids constructs

| Plasmid | Sequence |
| --- | --- |
| Lenti-EMMPRIN-2 | L: 5’- CGCGGATCCATGGCGGCTGCGCTGTTC -3’ |
|  | R: 5’- CCGGAATTCTCAGGAAGAGTTCCTCTG -3’ |
| Lenti-shRNA-Control (Scramble) | L:5’-CGCGTCCCCTTCTCCGAACGTGTCACGTTTCAAGAGAACGTGACACGTTCGGAGAATTTTTGGAAAT-3’ |
|  | R:5’-CGATTTCCAAAAATTCTCCGAACGTGTCACGTTCTCTTGAAACGTGACACGTTCGGAGAAGGGGA-3’ |
| Lenti-shRNA- EMMPRIN -2 | L:5’-CGCGTCGTCGTCAGAACACATCAACTTCAAGAGAGTTGATGTGTTCTGACGACTTTTTTAT -3’ |
|  | R:5’-CGATAAAAAAGTCGTCAGAACACATCAACTCTCTTGAAGTTGATGTGTTCTGACGACGA -3’ |
| Lenti-shRNA-Cathepsin B | L:5’-CGCGTCGTGGCCTCTATGAATCCCATTCAAGAGATGGGATTCATAGAGGCCACTTTTTTAT-3’ |
|  | R:5’-CGATAAAAAAGTGGCCTCTATGAATCCCATCTCTTGAATGGGATTCATAGAGGCCACGA-3’ |
